# PReS13-SPK-1472: Science of muscle training in inflammatory disease

**DOI:** 10.1186/1546-0096-11-S2-I9

**Published:** 2013-12-05

**Authors:** M Van Brussel

**Affiliations:** 1 Wilhelmina Children's Hospital, University Medical Center Utrecht, The Netherlands, Utrecht, The Netherlands

## 

Idiopathic inflammatory myopathies (IIMs) embody a heterogeneous group of conditions with a chronic autoimmune inflammatory process affecting a variety of muscle, skin and internal organs. IIMs are clinically characterized by (proximal) muscle weakness and decreased muscle endurance. Exercise training is increasingly utilized as a non-pharmacological intervention in the clinical management of pediatric patients with chronic inflammatory conditions; however, the efficacy, safety and effects on the course of the conditions should be topic of investigation. Clinicians attempting to prescribe exercise training in children with chronic inflammatory conditions face a dilemma. Exercise and physical training may encourage health e.g. by stimulating growth factors and tissue anabolism. In contrast, if sufficiently intense, exercise might stimulate inflammatory cytokines and lead to a catabolic state. Finding the optimal level of exercise in children and adolescents with an inflammatory condition can be difficult because the underlying disease can be associated with increased basal energy, malnutrition and inflammation, all of which promote tissue catabolism even at rest. Therefore, not only the efficacy and safety of training but also effects on the course of the conditions should be topic of investigation. The field of exercise physiology provides knowledge that might be of clinical importance when examining, testing and training muscle performance in children and adolescents with chronic inflammatory conditions.

## Disclosure of interest

None declared.

